# Improved Cytocompatibility and Reduced Calcification of Glutaraldehyde-Crosslinked Bovine Pericardium by Modification With Glutathione

**DOI:** 10.3389/fbioe.2022.844010

**Published:** 2022-05-19

**Authors:** Zhenlin Jiang, Zhongshi Wu, Dengpu Deng, Jiemin Li, Xiaoke Qi, Mingzhe Song, Yuhong Liu, Qiying Wu, Xinlong Xie, Zeguo Chen, Zhenjie Tang

**Affiliations:** ^1^ Department of Cardiovascular Surgery, The Second Xiangya Hospital, Central South University, Changsha, China; ^2^ NHC Key Laboratory of Birth Defect for Research and Prevention, Hunan Provincial Maternal and Child Health Care Hospital, Changsha, China; ^3^ Hunan Engineering Laboratory of Cardiovascular Biomaterials, Changsha, China

**Keywords:** glutaraldehyde crosslinking, glutathione detoxification, anti-calcification, cytocompatibility, biomaterial modification

## Abstract

Bioprosthetic heart valves (BHVs) used in clinics are fabricated via glutaraldehyde (GLUT) crosslinking, which results in cytotoxicity and causes eventual valve calcification after implantation into the human body; therefore, the average lifetime and application of BHVs are limited. To address these issues, the most commonly used method is modification with amino acids, such as glycine (GLY), which is proven to effectively reduce toxicity and calcification. In this study, we used the l-glutathione (GSH) in a new modification treatment based on GLUT-crosslinked bovine pericardium (BP) as the GLUT + GSH group, BPs crosslinked with GLUT as GLUT-BP (control group), and GLY modification based on GLUT-BP as the GLUT + GLY group. We evaluated the characteristics of BPs in different treatment groups in terms of biomechanical properties, cell compatibility, aldehyde group content detection, and the calcification content. Aldehyde group detection tests showed that the GSH can completely neutralize the residual aldehyde group of GLUT-BP. Compared with that of GLUT-BP, the endothelial cell proliferation rate of the GLUT + GSH group increased, while its hemolysis rate and the inflammatory response after implantation into the SD rat were reduced. The results show that GSH can effectively improve the cytocompatibility of the GLUT-BP tissue. In addition, the results of the uniaxial tensile test, thermal shrinkage temperature, histological and SEM evaluation, and enzyme digestion experiments proved that GSH did not affect the ECM stability and biomechanics of the GLUT-BP. The calcification level of GLUT-BP modified using GSH technology decreased by 80%, indicating that GSH can improve the anti-calcification performance of GLUT-BP. Compared with GLUT-GLY, GLUT + GSH yielded a higher cell proliferation rate and lower inflammatory response and calcification level. GSH can be used as a new type of anti-calcification agent in GLUT crosslinking biomaterials and is expected to expand the application domain for BHVs in the future.

## 1 Introduction

At present, the majority of bioprosthetic heart valves (BHVs) in clinical use in traditional thoracotomy operations or the rapidly developing interventional stent implantation are fabricated via glutaraldehyde (GLUT) crosslinking (Leong et al., 2013; Tripi and Vyavahare, 2014; Lim et al., 2015a; Tam et al., 2017; Fioretta et al., 2019; Wang et al., 2019; Song et al., 2021). GLUT strengthens the connection between collagen molecules, improves the mechanical strength, and blocks the main immunogenicity by binding with the amino groups on collagen molecules (Nachlas et al., 2017; Bonetti et al., 2019). Interventional stent BHVs made using GLUT crosslinking are expected to emerge as the mainstream trend of valve replacement in the future. It offers the advantages of superior hemodynamic performance and non-requirement of lifelong anticoagulants as well as fewer complications, higher quality of life, and less surgical trauma compared to mechanical valves (Umashankar et al., 2011; Wang et al., 2019; Fioretta et al., 2019; Mirani et al., 2021). However, when implanted into the human body, BHVs slowly release the cytotoxic free aldehyde group, which eventually causes valve calcification. Moreover, the negatively charged aldehyde groups remaining on the material surface can electrostatically adsorb calcium ions and enrich them to form calcium nuclei that eventually lead to valve calcification (Umashankar et al., 2011; Mathapati et al., 2013; Lim et al., 2015b; Flameng et al., 2015; Lee et al., 2017; Tod and Dove, 2016; Shang et al., 2017; Lopez-Moya et al., 2018; Meuris et al., 2018; Mirani et al., 2021). Therefore, eliminating or blocking residual aldehyde groups is an important way to improve the anti-calcification performance of tissues (Lim et al., 2015b; Flameng et al., 2015; Tod and Dove, 2016; Shang et al., 2017; Lopez-Moya et al., 2018; Meuris et al., 2018). The most frequently used method to address the issue is amino acid modification (Jorge-Herrero et al., 1996; Bezuidenhout et al., 2009; Braile et al., 2011; Chang et al., 2011; Lee et al., 2017; Park et al., 2017; Shang et al., 2017; Meuris et al., 2018; Braile-Sternieri et al., 2020), such as GLY modification, to block free aldehyde groups. This method was proven to effectively reduce toxicity and calcification, but its long-term effects need to be further evaluated and verified (Jorge-Herrero et al., 1996; Lee et al., 2011; Lim et al., 2015a; Lim et al., 2015c).

l-glutathione (N(N-l-γ-glutamyl-L-cysteinyl) glycine, GSH) is a tripeptide composed of cysteine, glutamic acid, and glycine (Pastore et al., 2003). Matsufuji Y found that one molecule of GSH can bind up to four molecules of acetaldehyde (Matsufuji et al., 2013). Therefore, we infer that GSH may also combine and block residual aldehyde groups in the tissue crosslinked with GLUT to reduce its toxicity and calcification level.

In this study, we used GSH rinse as new technology to modify the GLUT-fixed bovine pericardium (BP), thereby addressing the issues of toxicity and calcification. To explore whether GSH can be used as a new modification technology, we compared this method with GLY modification, which is widely used to block residual aldehyde groups and reduce the toxicity and calcification of GLUT-fixed biomaterials after implantation in the body.

## 2 Materials and Methods

### 2.1 BP Harvest

Native BPs of healthy Chinese yellow cattle (*Bos taurus*) were obtained from a local slaughterhouse (Changsha, China) and washed 3 times with 0.9% zsterilized saline at 4°C. After the surrounding fat and connective tissue were completely removed, the BPs were preserved in phosphate-buffered saline (PBS) with 1% penicillin–streptomycin and brought back to the laboratory for treatment.

### 2.2 Decellularisation

Considering that the original cell components in BPs may interfere with the results of our study (Liao et al., 2008; Crapo et al., 2011; Collatusso et al., 2019), we used the following steps to gently remove the cell components from the BPs: immersion in 0.1% bromogeramine solution at 37°C for 30 min; rinsing with 0.25% Triton X-100 (Sigma, X100, United States) at 37°C and 120 rpm for 48 h in a constant-temperature air table concentrator, with the Triton X-100 solution being replaced every 12 h; shock bleaching with 3 U/mL DNase-I (Servicebio, G5043, China)/0.03 mg/ml RNase-A (Servicebio, G3413, China) at 37°C and 120 rpm for 24 h, with the solution being replaced every 12 h; rinsing with distilled water for 48 h, with the rinsing water being replaced every 8 h; finally, rinsing with PBS for 24 h and storage after solution replacement. DNA quantification and hematoxylin and eosin (HE, Servicebio, G1005, China) staining of BPs before and after the steps were evaluated to determine the effect of decellularisation (*n* = 4). The cellular DNA in the BPs before and after the decellularisation was extracted using TIANamp Genomic DNA Kit (TIANGEN, Beijing, China) respectively, and quantified by NanoDrop 2000 (Thermo Fisher Scientific, MA, United States), then normalized to the dry weight of BPs.

### 2.3 Fixation of BPs

Decellularized BPs were placed with 0.625% GLUT (Aladdin, G105906, China) in PBS (pH 7.4) at room temperature with slight shaking for 48 h, and the solution was replaced every 24 h. Thereafter, the BPs were treated using three different methods and are denoted as follows:

1) GLUT: as control.
2) GLUT + GLY: the initial treatment of BPs was identical to that of GLUT control, followed by treatment with 0.2 mol/L GLY (GEN-VIEW, FG149, United States) in PBS (pH 7.4) at 4 C for 24 h.
3) GLUT + GSH: the initial treatment of BPs was identical to that of GLUT control. Thereafter, the BPs were treated with 8 mmol/L GSH (Yeasen, G5931120, China) in PBS (pH 7.4) at 37 C for 24 h. We set up different concentrations (0, 1, 2, 4, and 8 mmol/L) of GSH to modify the GLUT-fixed BP and finally selected 8 mmol/L as the most suitable modified GSH concentration through the qualitative and quantitative analyses of the aldehyde functional group (the specific steps were taken as follows).

A total of 20 independent samples (derived from individual animals, *n* = 20) for each group experiment were tested in this study.

### 2.4 Aldehyde Functional Group Assay

We used the acetaldehyde dehydrogenase (ALDH)-based assay reported by Lopez-Moya’s group to detect the content of the aldehyde functional group (Lopez-Moya et al., 2018). The BPs were thoroughly rinsed, ground into powder, and weighed (*n* = 4). Subsequently, the BPs were added with PBS and ground into a homogenate using grinding beads. The NAD+ (Yeasen, N3914880, China) and ALDH (Sigma, A6338-250UN, United States) solutions were sequentially added to the homogenate, and the reaction proceeded at room temperature for 1 h. Afterward, the system was centrifuged at 12,000 rpm for 5–8 min, and the supernatant solution was obtained for measuring the absorbance at 340 nm measured by the microplate reader (Thermo Scientific, MA, United States). The standard concentration of the GLUT solution was used to establish a standard curve, and the concentration and content of the aldehyde group in BPs were calculated according to the standard curve.

To calculate the content of aldehyde groups in each group,

$$\text{Total}\;\text{content}\;\text{of}\;\text{aldehyde}\;\text{groups}/\text{weight}\;\text{of}\;{\text{BP}}_{\text{S}}=\text{\_\_}\mu \text{g/g}\text{.}$$

Schiff reagent (Phygene, PH0643, China), which can react with aldehyde groups to form new red or purple-red compounds, was used to determine the content of aldehyde groups (Meuris et al., 2018).

### 2.5 Thickness and Water Content

Water on the BP surface was absorbed using dry gauze, and the thickness of the BPs was measured using a spiral micrometer (*n* = 10). The BP samples were placed in a freeze dryer (ThermoElectron Corporation, Milford, Massachusetts, United States) for 48 h to fully remove the moisture and weighed using an electronic balance to record the weight changes before and after dehydration (*n* = 8).

### 2.6 Uniaxial Tensile Test

Uniaxial tensile test was conducted using an electronic universal tensile testing machine (model Instron 4310 testing machine, Instron, United States). Every BP sample was cut into a rectangular shape (10 mm × 50 mm) with a cutter and then soaked in PBS before testing (*n* = 10). The thickness of the samples was measured using a digital micrometer. To prevent the tissue from slipping, metallic clasps were used on both ends of the holder. Using a 100 N load cell, the BP samples were preloaded (∼0.1 N) and stretched at a stretching speed of 10 mm/min, and the stress–strain curve was measured until the samples broke. The Young’s modulus (YM), ultimate tensile stress (UTS), and tensile stress (TS) of the samples were recorded.

### 2.7 Tissue Thermal Shrinkage Temperature

The BP shrinkage temperature was measured using a shrinkage temperature tester (Sichuan Chengdu Dachengxing Digital System Co., Ltd., China). Sample strips (1 cm × 5 cm) were cut from the BPs (*n* = 10) and heated gently at 4 C/min within the range of 20°C–120 C. The tissue shrinkage temperature was derived from the most extreme value within the endothermic peak.

### 2.8 *In Vitro* Collagenase Degradation Test

BPs were rinsed with deionized water, freeze-dried, and weighed. Thereafter, tissue samples (*n* = 10) were placed in 0.5 ml of 50 cdu/mL type I collagenase (Sigma, C2674-1G, United States), incubated at 37 C for 24 h, shaken at 120 rpm on an orbital shaker, washed with deionized water for 30 min, lyophilized, and weighed. The weight loss percentage of BPs was calculated as weight loss percentage = (weight before enzyme degradation − weight after enzyme degradation)/(weight before enzyme degradation) × 100%.

### 2.9 Cytotoxicity Test

Cytotoxicity testing was carried out according to the standard cytotoxicity test (Xu et al., 2017). Human umbilical vein endothelial cells (HUVECs) (EHY926, ATCC, United States) were cultured in a high glucose Dulbecco’s Modified Eagle Medium (DMEM) with 10% foetal bovine serum (FBS) (DMEM +10% FBS, Servicebio, G4510, China) at 37 C and under 5% CO_2_. 0.25% trypsin (Solarbo, T1350, China) was used to separate the cells to obtain cell suspension. Afterward, BPs were sterilized by γ radiation sterilization at a dose of 25 KGy, washed with sterile PBS (pH 7.4) 3 times, and soaked in (DMEM+10% FBS) culture media at 37 C for 24 h at a density of 2.5 ml/cm^2^. The sample leach liquor was collected and mixed with the culture media (DMEM +10% FBS) in a 1:1 ratio to prepare the corresponding cell culture media for each BP group. The culture media (DMEM +10% FBS) was designated as the blank control, and the leach liquor from the fresh BPs was denoted as the negative control group. Thereafter, The HUVECs were cultured by each group’s culture media for a further 1, 3, and 5 days at 37°C in a 96-well tissue culture plate, and then culture media was subsequently discarded. Each well was added with mitochondrial metabolic (MTT, Servicebio, G4104-1, China), incubated for 4–6 h, then added with dimethylsuphoxide (DMSO, Servicebio, G4104-2, China), and allowed to react for 20 min. The absorbance at 570 nm was measured using a microplate reader (Thermo Scientific, MA, United States). Each group was allocated three holes and cultured 6 times repeatedly.

The cell proliferation rate of BPs was calculated using the following equation (Wang et al., 2020):

$$\text{Cell}\;\text{proliferation}\;\text{rate}\;\left(\text{CPR}\right)\%\;=\left({\text{OD}}_{5}{-\text{OD}}_{1}\right)*100/{\text{OD}}_{1},$$

where OD_1_ and OD_5_ were the optical density on days 1 and 5, respectively.

### 2.10 Cell Proliferation Test

The samples were of 0.5 × 0.5 cm2 cut from BPs and immersed in 60% ethanol for overnight γ radiation sterilization. Thereafter, the samples were placed into a 48-well tissue culture plate and soaked in culture media (DMEM +10% FBS) overnight. The culture media (DMEM +10% FBS) was set as the blank control, while the group (fresh BPs) was designated as the negative control group. Human umbilical vein endothelial cells (HUVECs) (EHY926, ATCC, United States) were cultured on the surfaces of the BPs samples at a density of 2 × 10^4^ cells/ml in a constant-temperature cell incubator (37°C, 5% CO_2_) for 1, 3, and 5 days. The fluorescent 6-CDCFDA (Carboxy-2′,7′-dichlorofluorescein diacetate, Yuhengbio, C4096, China) was used to stain live cells, and cell growth was observed under a fluorescent microscope. Each group was assigned three holes and cultured 6 times repeatedly. ImageJ was used to calculate the cell count on the BP surface (Wang et al., 2020).

## 3 Hemolysis Test

Fresh anticoagulant blood was drawn from the ear vein of New Zealand rabbits (20 weeks old, 2.5–2.8kg, male, *n* = 4, HUNAN TAIPING SHENGWU KEJI YOUXIAN GONGS, China) and mixed with 3.8% sodium citrate at a ratio of 9:1 (v/v). Thereafter, anticoagulant-added blood and normal saline were obtained at a ratio of 4:5 (v/v) and prepared as diluted blood for subsequent use. The material was cut into a round sample with a diameter of ∼10 mm, rinsed with deionized water 3 times, placed in a 15 ml centrifuge tube, and added with 10 ml physiological salines. Subsequently, the samples were placed in a 37 C constant-temperature water bath for 30 min, and 0.2 ml of diluted rabbit blood was added to each tube. A mixture of 10 ml deionized water and 0.2 ml diluted blood was assigned as the positive control, and 10 ml of normal saline was the negative control. All centrifuge tubes were incubated at 37 C for 1 h and then centrifuged at 1,200 rpm for 5 min. The supernatant liquid was transferred to a cuvette, and the absorbance value at 545 nm was measured using a spectrophotometer. Each sample was measured 3 times.

The formula for calculating the hemolysis rate is as follows:

Haemolysis rate (HR)=(absorbance of sample−absorbance of negative control)/(absorbance of positive control−absorbance of negative control)×100%.

### 3.1 Histological Staining of BPs

The BPs were fixed in paraformaldehyde, embedded in paraffin, and sliced into 5 μm-thick strips. Several staining techniques, namely, hematoxylin and eosin (HE,Servicebio, G1005, China) staining for general morphology and cell nuclei, Masson’s trichrome (Servicebio, G1006, China) staining for collagen demonstration. The effects of each staining method on the BP matrix were observed under a light microscope.

### 3.2 SEM Analysis and Porosity Calculation

The BPs were fixed in 3% GLUT in PBS (pH 7.4) at 4 C for 2 h. After fixation, the samples were washed with PBS for 30 min, dehydrated by an ethanol series up to 100% ethanol, and dried at −55 C for 24 h in a freeze dryer. After drying, the sample was carefully mounted on an aluminium stub using a double-sided carbon tape (3 M Scotch, St. Paul, Minnesota, United States). The samples were subsequently placed into the chamber of a sputter coater (model SCD 005, Bal Tech Inc., Newport, Minnesota, United States) and sprayed with gold to make them conductive. The surface of BPs was scanned using a scanning electron microscope (Model Quanta 250 FEG, FEI Company, Czech Republic). ImageJ was used to calculate the porosity of BPs.

### 3.3 SD Rat Subdermal Implantation

All animal experiments in our study were authorized by the Animal Experiment Ethics Committee of the Second Xiangya Hospital of Central South University and the Authority for Animal Protection. All animal experiments complied with the ARRIVE guidelines and were performed in accordance with the United Kingdom Animal (Scientific Procedures) Act 1986 and its associated guidelines.

To prepare the samples for implantation, the BPs (cut into 1 cm × 1 cm squares, *n* = 10) were sterilized by γ radiation and rinsed with 50 ml sterile PBS. Male Sprague Dawley (SD) rats (4 weeks old, SJA LABORATORY ANIMAL CO. LTD, China)with weights ranging between 150 and 200 g (*n* = 15) were injected intraperitoneally with 1% pentobarbital (0.4–0.6 ml/kg). A surgical incision with two subcutaneous pockets on both sides was made on the back of each SD rat. A pericardium tablet was placed into each subcutaneous pocket and then fixed by polypropylene 4–0. Finally, the incisions on each SD rat were sutured using 7# silk thread. The specimens were extracted with the fiber capsule after 8 weeks of implantation. Half of the explants were retained for histological examination and immunohistological analysis. The specimens were frozen and weighed, and their calcium content was analyzed using the inductive ion coupling method (model ICP7400, Thermo Fisher, Waltham, Massachusetts, United States).

### 3.4 Calcium Content Analysis

The BPs were lyophilized, weighed, and solubilized in 1 ml 6 N HCl at 60 C for 4 h. After centrifugation at 12,000 rpm for 5 min, the supernatant was collected and diluted for 10 times in distilled water as the test solution (*n* = 10). The standard curve of the calcium ion concentration was plotted using the Ca^2+^ solution standard at 0, 2, 5, 10, and 20 μg/ml. Inductive ion coupling (model ICP7400, Thermo Fisher, Waltham, Massachusetts, United States) was used to determine the calcium content in the solution. The dry tissue weight was standardized, and the dilution ratio is used to calculate the calcium content.

### 3.5 Histological and Immunohistological Analysis of Implanted BP

The BP explants were taken out after 8 weeks, fixed with paraformaldehyde, ehydrated, and embedded in paraffin. Paraffin sections were stained for optical microscope analysis. hematoxylin and eosin (HE, Servicebio, G1005, China) staining was used to observe the overall condition of cells and specimens, while Masson’s trichrome (Servicebio, G1006, China) and Von Kossa (Servicebio, G1043,China) staining were used to examine the distribution of collagen fibers and calcium deposition in implanted samples, respectively. Rabbit anti-rat CD68 antibody (Servicebio Technology Co. Ltd., Wuhan, China; dilution 1:500) was used to label M0 macrophages (brown), while rabbit anti-rat CD3 antibody (Abcam, Cambridge, United Kingdom; dilution 1:500) was used to label T cells (brown).

### 3.6 Statistical Analysis

All experiments were performed a minimum of 3 times, and the results were represented as mean ± standard deviation. Statistical significance was determined using a one-way analysis of variance for the comparison of multiple groups. Asterisks (*) in figures indicate statistical significance (*p < 0.05, **p < 0.01, ***p < 0.001, and ****p < 0.0001, while ns represents no significant difference).

## 4 Results

### 4.1 Evaluation of Decellularisation Effect

**FIGURE 1:**
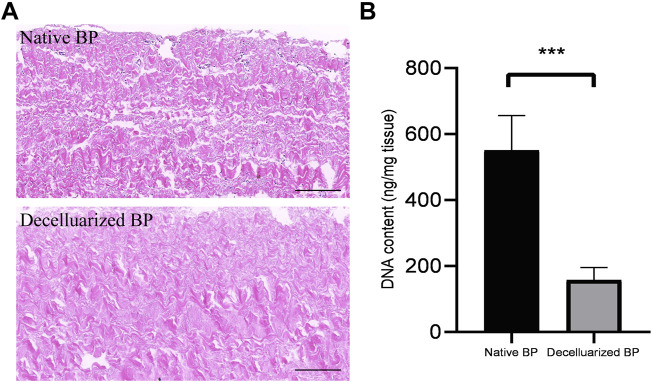
Validation of the decellularisation process of the borcine pericardiums (BPs) **(A)** HE staining of native BP (Fig.1A,top) and decellularized BP **(A)**. The black bar indicates 100 um **(B)** Quantitative determination of DNA in native BP and decellularized BP (*n* = 4; mean values ±s.d.∗*p* < 0.05; ∗∗*p* < 0.01; ∗∗∗*p* < 0.001; ns represents no significant difference).

The BPs were subjected to histological examination and DNA quantification before and after decellularisation to study the degree of cell removal. HE staining showed the nucleus and cell structure, which were basically not observed in BPs after decellularisation (Figure 1). The DNA quantitative results showed that decellularisation reduced the DNA content from 606.40 ± 57.50 ng/mg dry weight to 138.89 ± 26.29 ng/mg dry weight. This finding implies that our decellularisation procedure removed the DNA content ratio by more than 80% (*n* = 4, *p <* 0.001).

### 4.2 Qualitative and Quantitative Analyses of Aldehyde Groups

**FIGURE 2:**
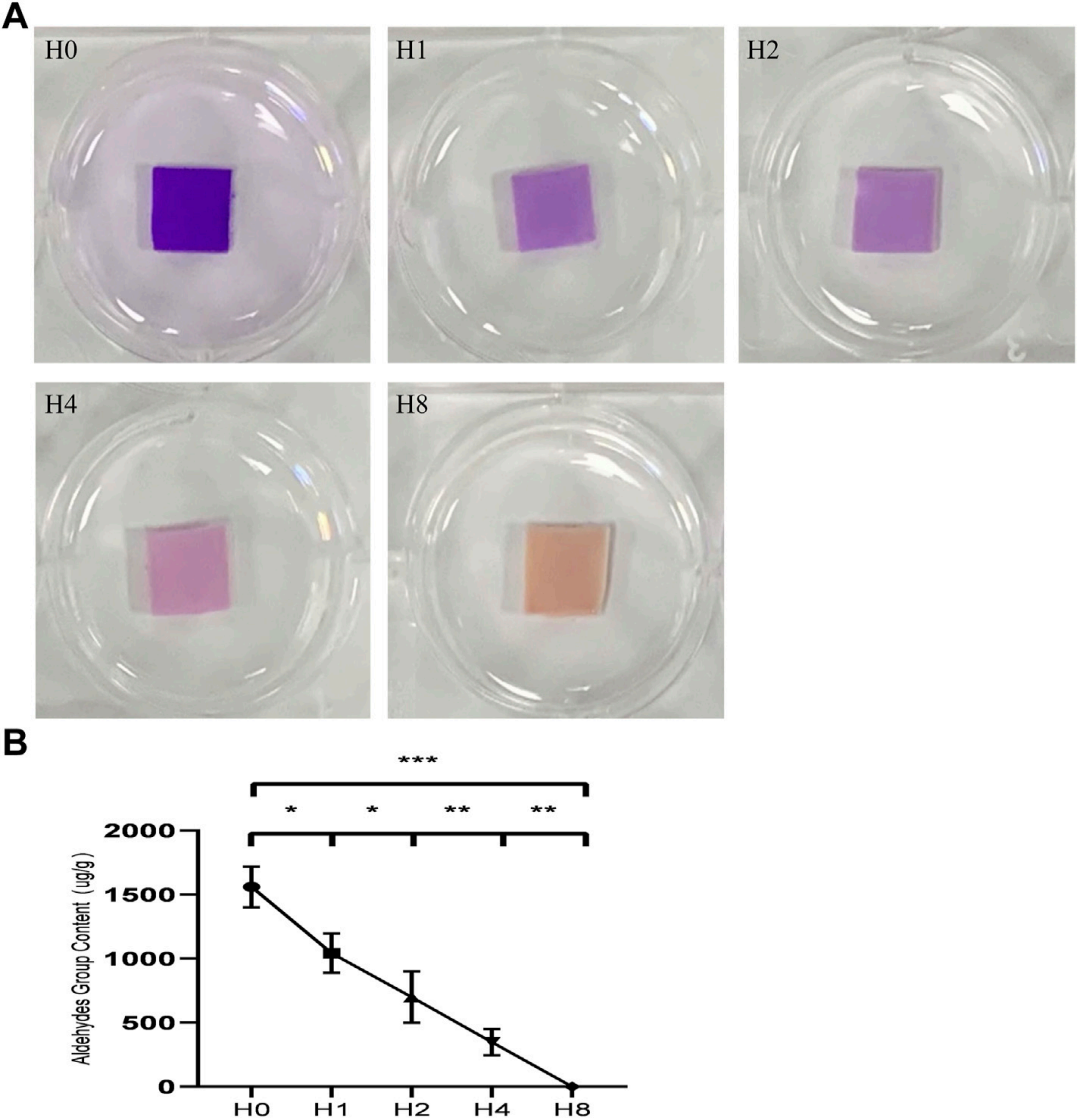
Qualitative and quantitative detection of aldehyde groups: **(A)**: results of different treatments of BPs stained with Schiff reagent, The color intensity is positively correlated with the content of aldehyde groups after the color reaction, The conditions of each group are as follows: H0:GLUT treated group; H1:GLUT+1 mmol/L GSH treated group; H2:GLUT+2 mmol/L GSH treated group; H4:GLUT+4 mmol/L GSH treated group; H8:GLUT+8 mmol/L GSH treated group; **(B)** is the qualitative content of aldehyde groups from different BP groups respectively (*n* = 4; ∗*p* < 0.05; ∗∗*p* < 0.01; ∗∗∗*p* < 0.001; ns represents no significant difference).

We used qualitative and quantitative aldehyde group experiments to evaluate the effect of GSH on blocking aldehyde groups. Schiff reagent can react with aldehyde groups to form new compounds, which are indicated by a red or purple color. The resulting compounds are stable and do not fade even in excess sulfurous acid, and their color depth after the reaction is positively correlated with the content of aldehyde groups. The results (Figure 2) show that with the increasing GSH concentration, the depth of the shade of the BP sample gradually becomes lighter, indicating a gradual decrease in the aldehyde group content. When GSH was not used, the aldehyde group content on the surface of GLUT-BP was 1,560.39 ± 159.55 μg/g. For GSH modification at concentrations of 1, 2, and 4 mmol/L, the contents of the aldehyde group are 1,043.00 ± 153.02, 699.88 ± 200.88, and 347.53 ± 101.61 μg/g, respectively (Figure 2B, *n* = 4, *p* < 0.05). When 8 mmol/L GSH was used for modification, the content of aldehyde groups decreased close to zero, indicating that the free aldehyde groups on GLUT-BP have been fully integrated into GSH. Therefore, 8 mmol/L was selected as the appropriate concentration for GSH modification in this experiment.

### 4.3 ECM Stability and Biomechanics

#### 4.3.1 Mechanical Uniaxial Tensile Test

**FIGURE 3:**
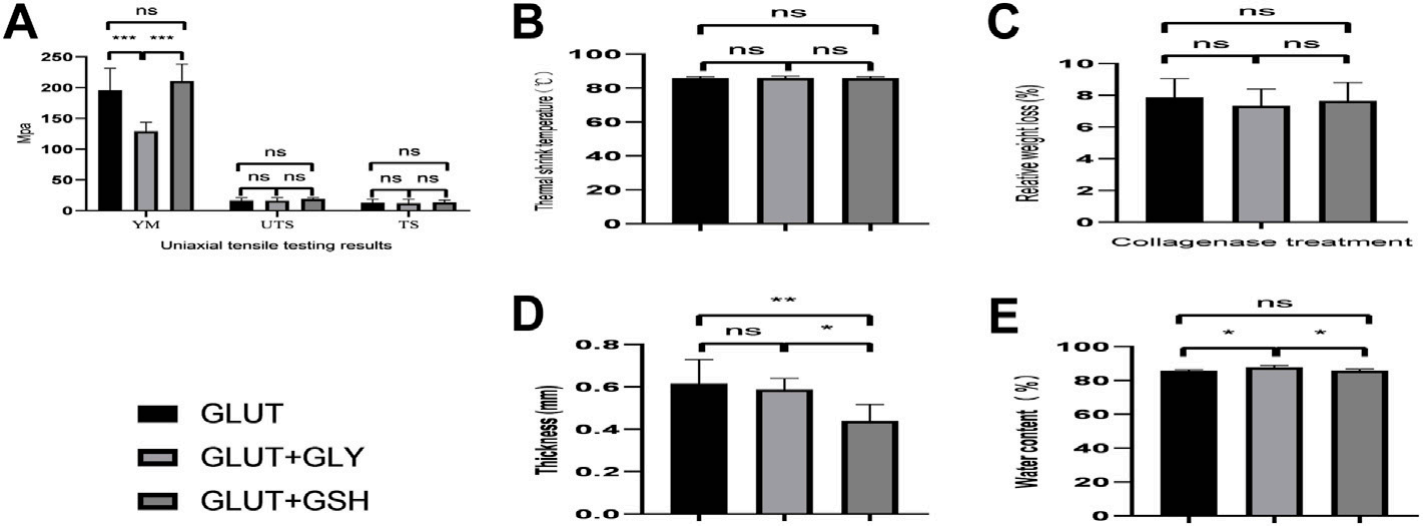
Biomechanical properties of BP from GLUT-BP, GLUT + GSH, and GLUT + GLY. **(A)** is mechanical properties of BP. Use the Young’s modulus (YM), ultimate tensile stress (UTS) and tensile stress (TS) to evaluate the mechanical properties of BPs. **(B)** is thermal shrinkage temperature, **(C)** is the BPs’ relative weight loss after collagenase treatment, **(D,E)** is thickness and water content of BPs from each group (*n* = 10; ∗*p* < 0.05; ∗∗*p* < 0.01; ∗∗∗*p* < 0.001; ns represents no significant difference).

According to the mechanical characteristic comparison of the two groups (Figure 3A), the YM, UTS, and TS of the GLUT + GSH group are not significantly different from those of the GLUT crosslinked group. However, the YM of GLUT + GLY is significantly lower than that of GLUT-BP (*n* = 10, *p* < 0.001).

#### 4.3.2 Thermal Shrinkage Temperature

We compared the thermal shrinkage temperature of BPs (Figure 3B). No statistical difference is found between the groups, indicating that GSH treatment does not affect the original strength and mechanical properties of the GLUT crosslinked tissue. This finding demonstrates that treatment with GLY or GSH has no effect on the thermal shrinkage temperature of BPs crosslinked by GLUT (*n* = 10, *p* > 0.05).

#### 4.3.3 Collagenase Degradation Test

Enzymatic hydrolysis experiments can be used to evaluate the strength of tissues. As observed from the results of collagen enzymatic hydrolysis experiments (Figure 3C), treatment with either GLY or GSH shows no statistically different effect on GLUT-BP (*n* = 5, *p* > 0.05). This result indicates that the two modifications are not significantly different, with both treatments having no effect on the GLUT crosslinking collagen.

#### 4.3.4 Water Content and Thickness

The thickness and water content of GLUT + GLY BPs (0.59 ± 0.53 mm, 87.82% ± 1.10%) significantly increased compared to those of GLUT-BP (0.62 ± 0.11 mm, 85.74% ± 0.63%; Figures 3D,E, *n* = 6, *p* < 0.05). Meanwhile, BPs treated with GLUT + GSH (85.78% ± 0.94%) showed no difference compared with the GLUT-BP group (Figures 3D,E, *n* = 6, *p* > 0.05).

### 4.4 Material Properties and Microstructure

#### 4.4.1 Histological Staining and SEM Analysis

**FIGURE 4:**
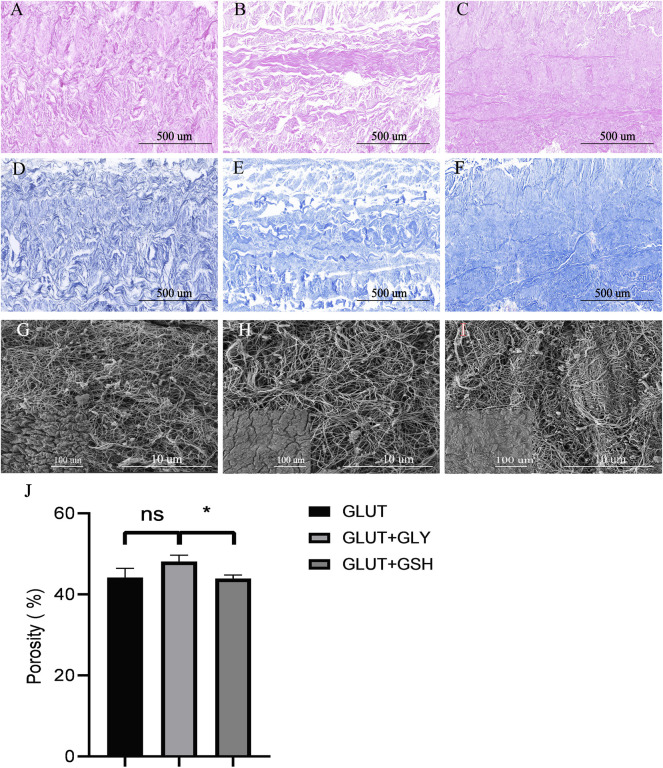
Histological staining (HE staining、Masson staining)and SEM for ECM evaluation of BPs from GLUT-BP **(A,D)**, GLUT + GLY **(B,E)**, GLUT + GSH **(C, F)**, The black bar indicates 500 um. The **(G,H,I)** is the SEM of GLUT **(A)**, GLUT + GLY **(B)**, GLUT + GSH **(C)** respectively, Each group shows a large picture (10.0kv, Scale bars = 10um) and a small picture on the bottom left corner (1.0kv, Scale bars = 100um). **(J)** is the porosity of BPs from each group (*n* = 4; ∗*p* < 0.05; ∗∗*p* < 0.01; ∗∗∗*p* < 0.001; ns represents no significant difference).

By combining the results of histological staining and the electron microscopy images of the microstructure of the BPs (Figure 4), we found that the microscopic fiber structure of GLUT-BP modified by GSH remained compact, and its porosity is not significantly different from that of GLUT-BP. By contrast, the structure of GLUT-BP after GLY treatment is relatively changed, and the porosity of GLUT + GLY is significantly lower than that of GLUT + GSH (Figure 4J, *n* = 5, *p* < 0.05). These findings indicate that the tissue modified by GSH can retain the microstructure of the extracellular matrix of GLUT-BP to a higher degree compared to that modified by GLY.

### 4.5 Cytocompatibility

#### 4.5.1 Cell Proliferation and Morphology Assays

**FIGURE 5:**
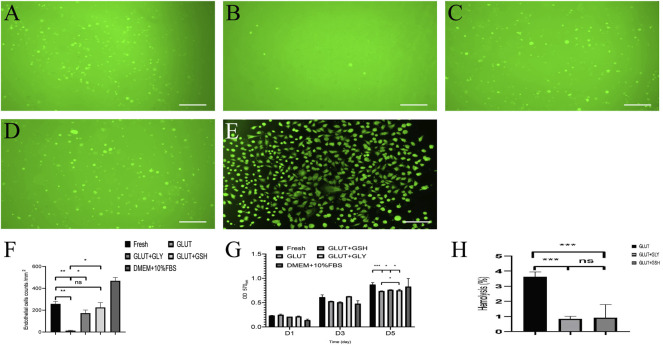
Cytocompatibility of BPs *in vitro*, A-E is the cell fluorescence staining of Fresh-BP **(A)**, GLUT-BP **(B)**, GLUT + GLY **(C)**, GLUT + GSH **(D)**, and DMEM+10%FBS **(E)** by 6-CDCFDA after 5 days of culture on the BPs surface respectively. **(F)** is the count of endothelial cells of BPs. The cell proliferation rate (n = 4, mean values ±s.d.) of EAHY926 cells were determined using the MTT assay kit **(G)**. **(H)** is the hemolysis percentage of BPs (*n* = 4; ∗*p* < 0.05; ∗∗*p* < 0.01; ∗∗∗*p* < 0.001; ns represents no significant difference).

We used a cell proliferation test to evaluate the materials surface toxicity of BPs and whether it is convenient for cell adhesion. We cultured HUVECs on the surface of BPs (Figures 5A–E) and then counted their quantities as shown in Figure 5F. The HUVECs counts for the materials surface of Fresh BP, GLUT-BP, GLUT + GLY, and GLUT + GSH are 257.75 ± 23.53, 12.75 ± 2.87, 173 ± 28.72, and 225.25 ± 44.56 cells/mm^2^, respectively. The condition of cell proliferation and adhesion from GLUT + GSH is approximately equal to that of the Fresh BP (*n* = 4, *p* > 0.05), indicating that GSH treatment can significantly reduce the cytotoxicity of GLUT-BPs materials surface. In addition, the cell proliferation and adhesion performance of GSH are significantly stronger than that of GLY.

Furthermore, we used a cytotoxicity test to evaluate the overall toxicity of BPs materials. The cytotoxicity in leach liquor from BPs shows that (Figure 5G), the cell proliferation ability of GLUT-BP (CPR: 195%) was significantly lower than that of Fresh BP (CPR: 265%); by contrast, those of GLUT + GLY (CPR: 247%) and GLUT + GSH (CPR: 262%) significantly improved.

These test results prove that GLY and GSH can reduce the cytotoxicity and improve the cytocompatibility of GLUT-fixed BPs (Figure 5G, *p* < 0.05). Notably, the results of both cell proliferation tests and cytotoxicity showed no statistically significant difference between GLUT + GSH and Fresh BP (*n* = 4, *p* > 0.05).

#### 4.5.2 Hemolysis Test

Results of the hemolysis experiment (Figure 5H) show that the hemolysis rate of GLUT-BP is significantly reduced after treatment with GLY and GSH, with a statistically significant difference (*n* = 4, *p* < 0.05). According to these results, GLY and GSH can significantly improve the blood compatibility of GLUT-BP.

#### 4.5.3 *In Vivo* Biocompatibility Assay

**FIGURE 6:**
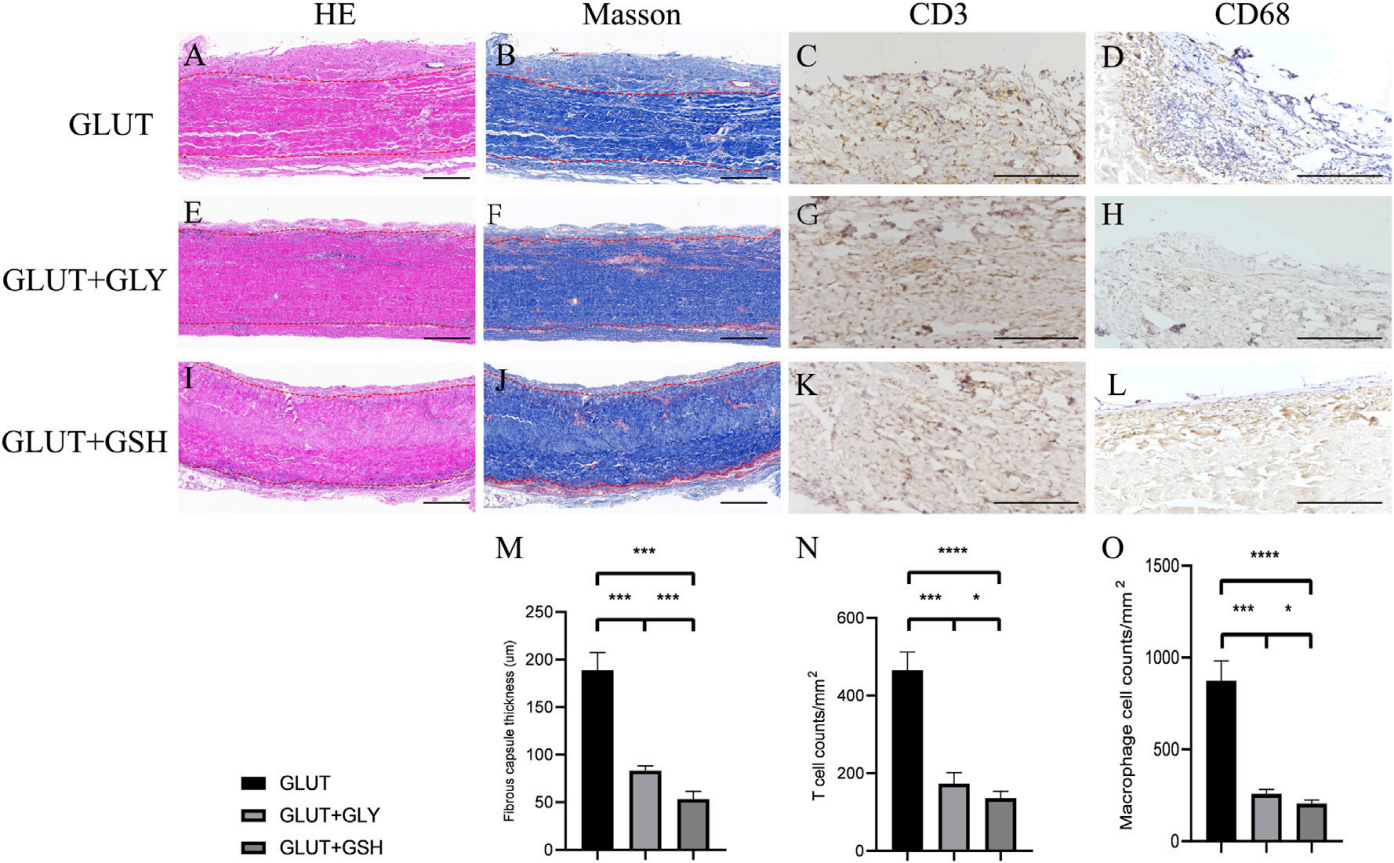
*In vivo* biocompatibility of BPs from GLUT-BP **(A–D)**, GLUT + GLY **(E–H)**, GLUT + GSH **(I–L)**. The **(M–O)** is the Fibrous capsule thickness and count of T cell and macrophage cell (*n* = 4; ∗*p* < 0.05; ∗∗*p* < 0.01; ∗∗∗*p* < 0.001; ∗∗∗∗*p* < 0.0001; ns represents no significant difference).

BPs were implanted subdermally in rats for 8 weeks and extracted for histological and immunohistological analyses. For GLUT-BP, GLUT + GSH, and GLUT + GLY, HE staining images revealed that inflammatory cells were recruited to the BP interface rather than migrating inside of the GLUT-BP implants after 8 weeks of implantation. The phenotype of inflammatory cells surrounding the implants was further analyzed using CD68 and CD3 markers, which can mark the macrophage and T cells, respectively. GLUT-BP evidently recruited more macrophage and T cells than GLUT + GLY and GLUT + GSH after 8 weeks of implantation (Figures 6D,H,L). The number of T and macrophage cells of GLUT + GSH was significantly lower than those of the other two groups (Figures 6C,G,K, *n* = 4, *p* < 0.05). Masson staining revealed that GLUT-BP was encapsulated with a dense collagenous capsule, whereas GLUT + GSH or GLUT + GLY was surrounded by a relatively looser and thinner collagenous capsule (Figures 6B,F,J). In all pathological stains, the collagen density surrounding GLUT was higher than that surrounding GLUT-GLY and GLUT + GSH (Figures 6B,F, and 6, *n* = 4, *p* < 0.05), thereby indicating that GLUT elicited a more severe chronic inflammatory response without treatment. Compared to GLUT + GLY, GLUT + GSH triggered a significantly lower inflammatory response (*n* = 4, *p* < 0.05). Based on the counts and statistical data of T and macrophage cells and the thickness of the fibrous capsules, we concluded that although GLY modification can reduce the degree of the inflammatory response of GLUT-BPs, GSH modification has a better effect in reducing the inflammatory response after implantation.

### 4.6 *In Vivo* Anti-Calcification Assay

**FIGURE 7:**
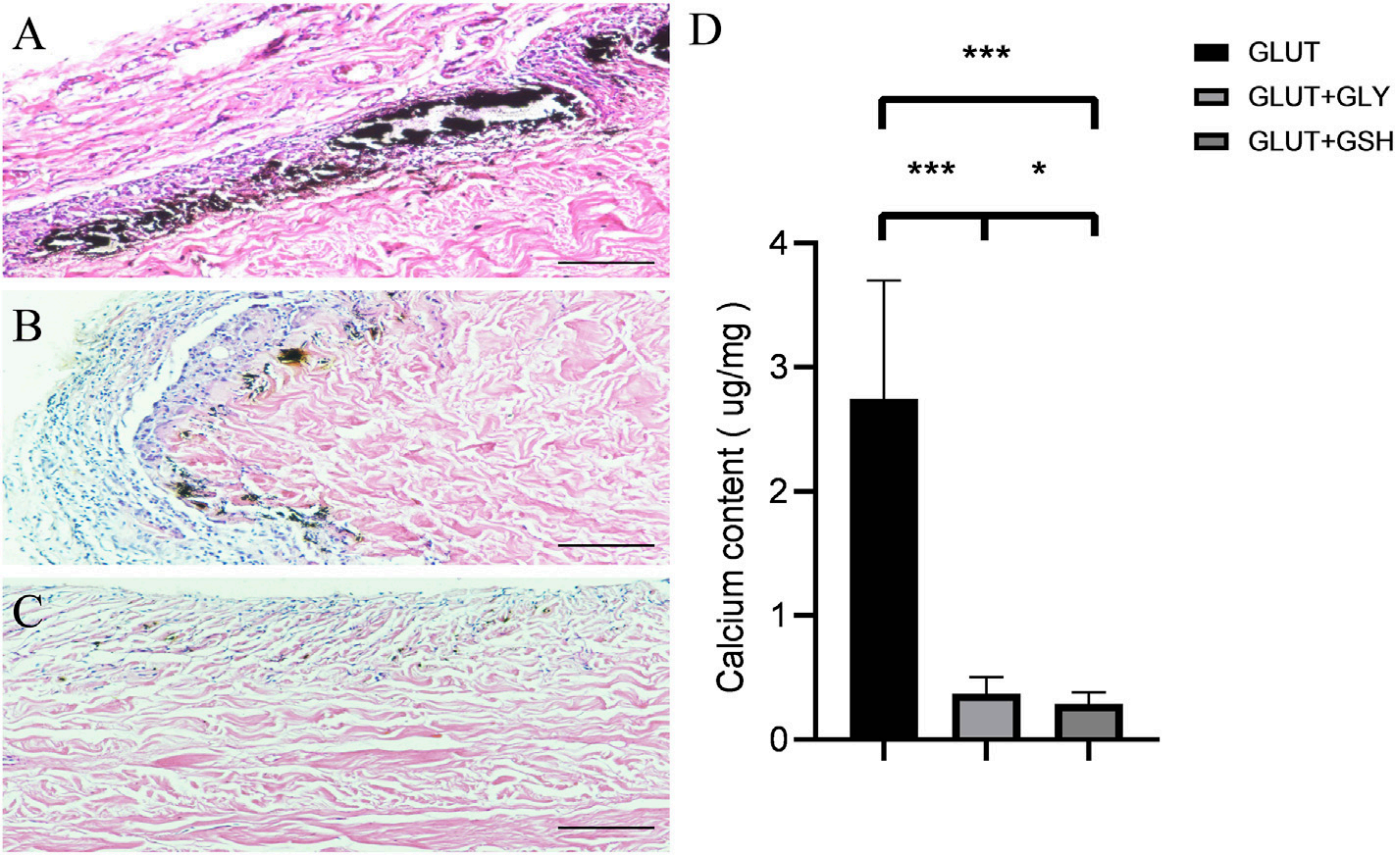
After 8 weeks of subcutaneous implantation in the rat, the BPs were taken out for Von kossa calcium staining and calcium content determination. The **(A–C)** is the calcium staining in the GLUT group and the GLUT + GLY and GLUT + GSH treatment groups. The black scale bars are 500 um. The Fig.7 **(D)** is the calcium content determination in the GLUT group and the GLUT + GLY and GLUT + GSH (*n* = 10; ∗*p* < 0.05; ∗∗*p* < 0.01; ∗∗∗*p* < 0.001,∗∗∗∗*p* < 0.0001).

Figure 7 shows the results of the *in vivo* experiments on rats with different treatment tissues implanted subcutaneously for 8 weeks Figures 7A–C show calcium staining in GLUT-BP, GLUT + GLY, and GLUT + GSH, respectively. No significant difference is found between the tissue morphologies of BPs before implantation. In the calcium staining image of the GLUT group, calcification foci were visible; by contrast, no calcification foci were found in GSH. Figure 7D shows the determination of the calcium content in the subcutaneously embedded specimens of the two groups. The results show the calcification contents for GLUT (2.68 ± 0.84 μg/mg, *n* = 10), GLUT + GSH (0.27 ± 0.08 μg/mg, *n* = 10), and GLUT + GLY (0.39 ± 0.11 μg/mg, *n* = 10). GLY and GSH treatment resulted in a significantly lower calcium content relative to that of GLUT-BP (*n* = 10, *p* < 0.001), with GSH exhibiting a better anti-calcification performance than GLY (*n* = 10, *p* < 0.05).

## 5 Discussion

The main reason for the calcification of BHVs fixed by GLUT is the presence of aldehyde groups on the BHV surface; by reducing the content of aldehyde groups on the BHV surface, the degree of BHV calcification can be effectively attenuated (Tod and Dove, 2016; Shang et al., 2017; Lopez-Moya et al., 2018; Fioretta et al., 2021). In this study, we selected GSH as a novel modification agent and compared it with GLY in terms of the qualitative and quantitative detection of aldehyde groups, biocompatibility, and anti-calcification performance.

The qualitative and quantitative tests for aldehyde group detection showed that rinsing GLUT-BP with 8 mmol/L GSH at 37 C for 24 h can completely block the residual aldehyde groups and remove the toxicity of GLUT-BP. Our results also showed that as the concentration of GSH increased, the content of aldehyde groups gradually decreased until it was eliminated, further demonstrating the effectiveness of GSH in blocking aldehyde groups. In this study, we referred to relevant studies for the modification conditions of GLY (Lim et al., 2015a; Park et al., 2017). The molar concentration of GSH used is 1/25 of that of GLY, but GSH achieved a better effect. We speculated that because GSH has more active binding sites for aldehyde groups compared to GLY, with 4 binding sites in each GSH molecule (Matsufuji et al., 2013), the ability of GSH to bind to aldehyde groups is consequently stronger than that of GLY.

We explored cytocompatibility in terms of cell toxicity, hemolysis rate, and inflammation *in vivo* and *in vitro*. For cell toxicity, we counted and compared the cell attachment and proliferation of GLUT-BP, GLUT + GLY, and GLUT + GSH, with Fresh BP as the negative control. With GSH or GLY for GLUT-BP modification, endothelial cells proliferated well after 5 days of seeding. By contrast, GLUT-fixed BPs exhibited substantially poor cell adhesion and proliferation. This finding shows that modification by GSH and GLY can effectively eliminate cytotoxicity and improve the biocompatibility of GLUT-BP. Moreover, compared with GLUT + GLY, GLUT + GSH provides a better condition for cell adhesion and proliferation, with no statistical difference compared with Fresh BP (*p* > 0.05). This indicates that after GSH modification, the cytocompatibility of GLUT-BP is equivalent to that of the fresh BPs. Results of the hemolysis experiment in this study also show the same trend. Although the hemolysis rates of GLUT, GLUT + GSH, and GLUT + GSH are relatively low, modification using GSH and GLY has yielded a statistically significant reduction in the hemolysis rate. The BHV is in direct contact with the circulating blood after transplantation in the human body; considering the long-term cumulative toxicity effects, we believe that the reduction effect of GSH and GLY on the hemolysis rate of GLUT-BP is of particular importance. After confirming our findings with *in vitro* experiments, we also carried out *in vivo* experiments via SD rat subdermal implantation. The thickness of the fibrous capsule and the number of inflammatory cells can clearly reflect the degree of the inflammatory reaction of implanted BP *in vivo*. As observed from the results of staining and cell count *in vivo* experiments, the thickness of fibrous capsules and the number of inflammatory cells with GSH treatment are significantly lower than those in GLUT and are superior to those with GLY treatment. GSH has been reported to reduce the expression of inflammatory factors and inhibit the production and aggregation of inflammatory cells (Rahman et al., 2005; Perricone et al., 2009; Bajic et al., 2019), and this point is confirmed in our study. We also analyzed the differences in cell compatibility between GLY and GSH. Although both GLY and GSH can block aldehyde groups, GSH offers strong reducibility and anti-inflammatory properties as a strong reductant that GLY does not possess. As a reductant, GSH can eliminate intracellular reactive oxygen species (ROS) and reduce NF-κB retained in the cytoplasm, preventing it from entering the nucleus and inducing the transcriptional expression of pro-inflammatory cytokines, such as IL-1, IL-6, and TNF-α (Tewes et al., 1997; Rahman, 1999). GLY does not possess these capabilities.

Through a series of biomechanical performance evaluations, such as anti-enzymatic performance and anti-calcification performance evaluations, our study showed that the use of GSH modification can improve the compatibility and anti-calcification performance without affecting the mechanical properties and the organizational strength of extracellular matrix crosslinked by GLUT. However, we observed a significant deterioration in the mechanical properties of GLUT + GLY, such as YM. We tested and compared the water content and thickness of each BP group and observed an increased thickness and water content of GLUT + GLY. We speculate that the addition of GLY significantly improves the hydrophilicity of GLUT-BP, thereby increasing the water content of the BPs and, consequently, its thickness. The BPs become swollen, eventually leading to a decrease in mechanical properties. Moreover, by calculating and comparing the porosities of the BPs of each group, we found that the porosity of GLUT + GLY was significantly lower than that of GLUT + GSH. This verified that our GLY modification caused the GLUT-BP to become looser, and this result was consistent with the data on the water content and thickness.

In the subcutaneous embedding in SD rats, the calcium content of BPs decreased by 70% with GSH treatment, and the anti-calcification performance of the material was significantly improved. The results with GSH were significantly different from those with GLY. The calcification level of GLUT + GSH is much lower than that of GLUT + GLY, indicating that GSH modification can be used as a new anti-calcification treatment technology for GLUT-fixed biological materials. GSH has been proven to reduce vascular calcification (Mody et al., 2001; Roman-Garcia et al., 2011; Patel et al., 2021), and our results corroborate these previous findings. We believe that the GSH reduction of the GLUT-BP calcification may follow various reasons. For example, GSH reduces the cytotoxicity of GLUT-BP and the inflammatory response in the rat body in our study. Furthermore, this modification technology does not affect the biomechanical properties of GLUT-BP. After GSH modification, GLUT-BP becomes a dense, stable, and excellent histocompatibility material. The combined effect of these factors causes a significant improvement in anti-calcification performance.

To the best of our knowledge, there is no relative report about the GSH used in GLUT modification internationally. Our study established an effective technology involving the use of GSH and was the first to report its use in blocking residual aldehyde groups by exploring concentration and reaction conditions. All these results showed that GSH can be used as a new mild, efficient modification agent for GLUT-fixed biomaterials.

## 6 Conclusion

GSH modification yields an excellent improvement in the cytocompatibility and anti-calcification performance for GLUT-fixed materials while maintaining their original biomechanical properties. It can replace GLY modification and be used as a novel anti-calcification modification technology for heterogeneous tissue materials crosslinked by GLUT. We believe that GSH will have a wider range of applications for GLUT-fixed biomaterials in the future.

### 7 Limitations of the Study

Although this study explored GSH as a novel modification technology and proved that GSH can effectively improve biocompatibility and anti-calcification ability, more research is needed to determine the treatment effect under long-term blood circulation *in vivo*, such as demonstrating the efficacy of this novel BHV treatment through accelerated *in vitro* fatigue tests. In addition, valves fabricated from GLUT + GSH need to be examined in large animal studies via implantation as cardiac valve substitutes to establish their function under blood contact and resistance to degradation and calcification. Through further understanding of this novel technology and optimized processing to produce GLUT crosslinked structures with excellent durability and biocompatibility, GLUT-fixed BHVs can offer a broad research and application prospect.

## Data Availability

The raw data supporting the conclusion of this article will be made available by the authors, without undue reservation.
